# Multifocal Breast Cancer in Young Women with Prolonged Contact between Their Breasts and Their Cellular Phones

**DOI:** 10.1155/2013/354682

**Published:** 2013-09-18

**Authors:** John G. West, Nimmi S. Kapoor, Shu-Yuan Liao, June W. Chen, Lisa Bailey, Robert A. Nagourney

**Affiliations:** ^1^Breastlink, Department of Surgery, 230 S. Main Street, Suite 100, Orange, CA 92868, USA; ^2^Department of Pathology, St. Joseph Hospital, University of California Irvine, 1100 West Stewart Drive, Orange, CA 92868-5600, USA; ^3^Breastlink, Department of Radiology, 230 S. Main Street, Suite 100, Orange, CA 92868, USA; ^4^Bay Area Breast Surgeons, Inc., Department of Surgery, 3300 Webster Street, Suite 212, Oakland, CA 94609, USA; ^5^Department of Obstetrics and Gynecology, Rational Therapeutics, University of California Irvine, Long Beach, CA, USA

## Abstract

Breast cancer occurring in women under the age of 40 is uncommon in the absence of family history or genetic predisposition, and prompts the exploration of other possible exposures or environmental risks. We report a case series of four young women—ages from 21 to 39—with multifocal invasive breast cancer that raises the concern of a possible association with nonionizing radiation of electromagnetic field exposures from cellular phones. All patients regularly carried their smartphones directly against their breasts in their brassieres for up to 10 hours a day, for several years, and developed tumors in areas of their breasts immediately underlying the phones. All patients had no family history of breast cancer, tested negative for BRCA1 and BRCA2, and had no other known breast cancer risks. Their breast imaging is reviewed, showing clustering of multiple tumor foci in the breast directly under the area of phone contact. Pathology of all four cases shows striking similarity; all tumors are hormone-positive, low-intermediate grade, having an extensive intraductal component, and all tumors have near identical morphology. These cases raise awareness to the lack of safety data of prolonged direct contact with cellular phones.

## 1. Case Reports

### 1.1. Case 1

**Figure 1:**
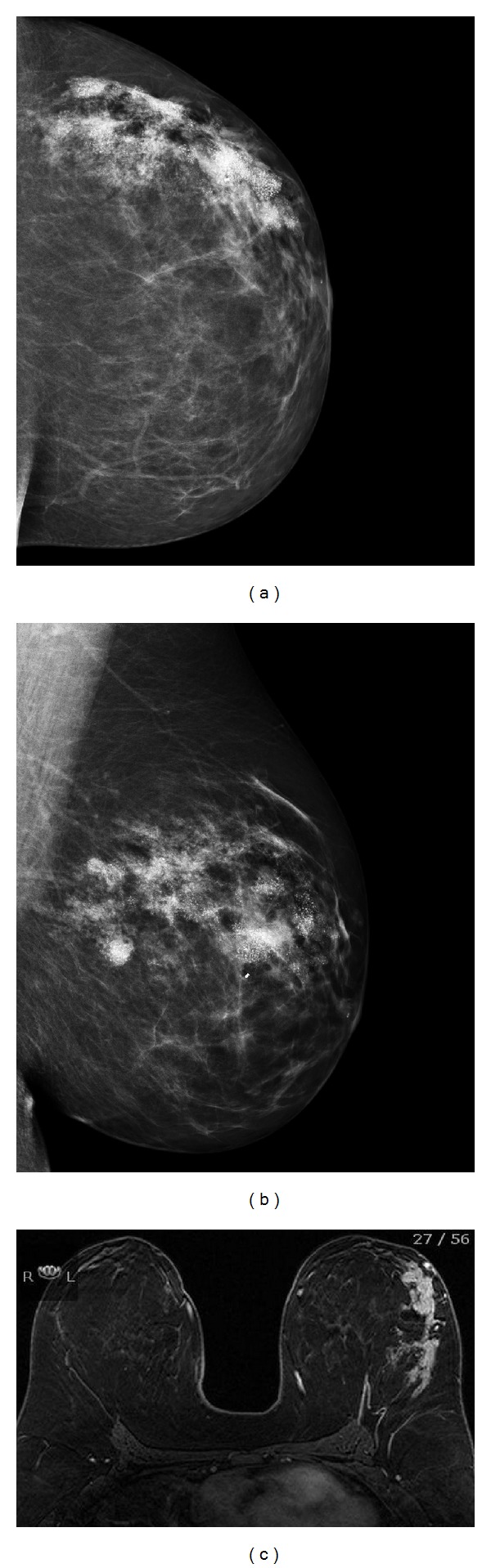
Representative imaging of patient in Case 1. Left mammogram showing clustered calcification corresponding to multiple sites of disease in craniocaudal (a) and mediolateral-oblique (b) projections. MRI showing extensive nonmass enhancement in the lateral hemisphere of the left breast in segmental distribution (c).

A 21-year-old female presented with left spontaneous bloody nipple discharge. Her history was notable for keeping her cellular phone tucked into her bra on the left side for several hours each day. Her mammogram showed extensive pleomorphic calcifications and densities from the retroareolar region to the chest wall spanning a length of 12 cm. A magnetic resonance image (MRI) showed extensive abnormal nonmass enhancement in a segmental distribution corresponding to changes seen on her mammogram (Figures 1(a)–1(c)). She was treated with mastectomy and pathology revealed extensive ductal carcinoma in situ (DCIS) with multifocal microinvasion. Sentinel lymph nodes were negative for metastatic disease.

### 1.2. Case 2

A 21-year-old female presented with a palpable breast mass in the area where her cellular phone was kept in direct contact with her left breast. She had been placing her cellular device in her bra for eight hours a day or longer for the past six years. Breast MRI demonstrated four distinct separate lesions ranging from 15 to 18 mm in diameter involving an extensive area of the upper hemisphere of the left breast. Pathology of her mastectomy showed multifocal invasive cancer with extensive DCIS. Two of nine axillary lymph nodes were positive for metastatic disease. Later studies found metastasis to the bone.

### 1.3. Case 3

A 33-year-old female presented with two palpable masses in the upper outer quadrant of her right breast directly underneath where her cellular phone was placed against her breast in her bra. She had been placing her cellular phone in her bra intermittently for eight years. In the two years prior to diagnosis she would routinely place her phone in her bra while jogging 3-4 times per week. During this time period she would use a global positioning system (GPS) application on her cellular phone to determine her location while jogging. MRI demonstrated at least six suspicious lesions spanning a length of 8 cm in the upper outer quadrant of the right breast. Mastectomy specimen showed extensive DCIS with multifocal invasion. A 5 mm metastasis was found in one sentinel lymph node.

### 1.4. Case 4

**Figure 2:**
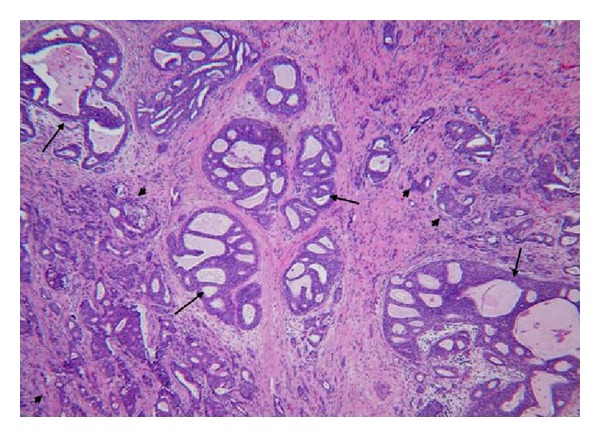
Representative histology of all four cases. There is extensive DCIS with cribriform configuration (arrow). The multiple foci of invasion (arrowhead) occur in between the DCIS (magnification ×100).

A 39-year-old female presented with three palpable breast masses in the area of cellular phone contact with her right breast. She had been placing her cellular phone in her bra while commuting and using a Bluetooth device to talk for hours each day for the past ten years. MRI demonstrated multiple mass-like and tubular areas of enhancement essentially involving the entire upper right breast from the 11 to 1 o'clock position. Mastectomy showed four separate invasive ductal carcinomas ranging from 1 to 3 cm in size with 10 cm of DCIS. Two of nine lymph nodes were positive for metastatic disease. Pathology of the insitu and invasive ductal carcinomas observed in all four cases shows striking similarity, and the representative histological figures are illustrated in Figure 2.

## 2. Discussion

The majority of breast cancer occurs sporadically in postmenopausal women with no family history of the disease. Breast cancer occurring in women in their 20s and 30s is uncommon, accounting for fewer than 5% of all breast cancer cases, and is often associated with a genetic predisposition [1]. These four cases of young women with sporadic, multifocal breast cancer bring forth the possibility of a relationship between prolonged direct skin contact with cellular phones and the development of breast cancer. To date there is insufficient laboratory or clinical evidence to establish a definite relationship between exposures to the electromagnetic radiation (EMR) emitted from cellular devices and the risk of developing cancer. Some studies have suggested that such a relationship exists, but larger and more robust studies have not been confirmatory [2–6]. Nonetheless, the International Agency for Research on Cancer has classified radiofrequency waves of the electromagnetic spectrum, the form of EMR that cellular devices emit, as a possible human carcinogen [7].

Cellular phones emit EMR in the microwave spectrum and produce both a thermal and nonthermal effect. The EMR emitted from cellular phones has insufficient energy to ionize molecules and is not capable of producing direct DNA damage as occurs with diagnostic and therapeutic radiation [2]. The primary thermal effect from cellular phones is the heating of tissue, which has controversial clinical significance [2, 8–10]. EMR emitted from cellular devices couples with the body to create currents within the tissue, potentially having an effect on cellular microenvironments [9]. A recent study using fluorodeoxyglucose injections and positron emission tomography concluded that exposure to radiofrequency waves within parts of the brain closest to the cellular phone antenna resulted in increased levels of glucosemetabolism, but the clinical significance of these findings is unknown [11].

One of the first clinical reports of a possible carcinogenic effect of exposure to EMR from cellular phones suggested that cellular phone users were at increased risk of developing brain cancers [5]. The largest and longest study of cellular phone use to date is the INTERPHONE study which included data from 13 countries [6]. This retrospective study could not identify a significant increase in risk of gliomas or meningiomas with the use of cellular phones. There were, however, indications of an increased risk of glioma at the highest exposure levels, but biases and error prevent a causal interpretation. The INTERPHONE study concluded that the possible effects of long-term, heavy use of mobile phones require further investigation. A more recent meta-analysis showed an association between gliomas and acoustic neuromas in ipsilateral users (using the phone on one side of the head most often or always) who were also heavy users of cellular phones, compared to nonusers [3]. Moreover, the risk of cancer was found highest in people with longest exposure and exposure that began before the age of 20. 

The issue of cellular phone exposure on male fertility has also been reported [12]. Both laboratory and clinical studies have demonstrated alterations in fertility, motility, and morphology in sperm exposed to EMR from cell phones. Similar reports of clinical responses resulting from exposure to cellular phone EMR have been made for changes in the blood brain barrier and cognition, but attempts to confirm these findings have been inconsistent [13–15].

The data collected from the majority of the aforementioned cellular phone studies was from the early 2000s. Since that time, cellular phone usage has continued to increase, with over 303 million subscribers to cellular phone service in the United States alone in 2011 and almost six billion subscribers worldwide [16]. This is triple the number of reported users in 2000. Children and young adults are now more likely to be using mobile devices and are among some of the heaviest users [17]. This group is potentially at greatest risk of harm from EMR, as dividing tissue, such as that occurring in prepubertal breast buds, is more prone to the adverse effects of radiation [18].

Current cellular phone safety regulations were established in the United States by the Federal Communication Commission (FCC) in 1996 [19]. The regulations were based on studies which measured the level of EMR penetrating the plexiglass head of a simulated 200 pound man. The studies were designed to measure the specific absorption rate (SAR) which is a measure of the rate at which energy is absorbed by the body when exposed to cellular phone EMR. The FCC set an exposure limit of 1.6 watts per kilogram of tissue. Any cellular phone functioning below this limit is considered to be safe. The duration of exposure during a SAR test is only 30 minutes and does not reflect the total amount of EMR exposure consumers experience with more prolonged exposure. Furthermore, FCC guidelines do not address the issue of risks associated with direct skin contact with cellularphones. This is a critical issue, as the long-term consequence of the direct thermal effect of EMR on developing breast tissue for extended duration has not been documented. In addition, unlike older cellular devices, smartphones have the ability to regularly transmit information, sending and receiving an intermittent signal even when the user of the device is not actively handling it. The accumulation of this passive exposure to EMR is also not well studied. 

Although the FCC has not addressed the issue of skin contact, cellular phone manufacturers typically place a warning in their manuals stating that direct contact with the skin should be avoided. For example, the iPhone 4 user manual advises to keep the phone 1.5 cm or more away from the body [20]. Similarly, the safety manual for the BlackBerry Bold Smartphone recommends using an approved holster to carry the phone and to keep the phone 15 mm away from the body when the device is transmitting [21].

This series of four young women with cellular phone-related breast cancer is noteworthy, but caution must be exercised in drawing any conclusion from our small sample. Millions of women are using cellular devices, and it is predictable that rare events will occur. From this small case series, one cannot infer causality but can only consider association. Additionally, no data is available on the number of women who place their cellular phones in contact with their breast and do not develop breast cancer. Finally, the duration of exposure and the location of placement of the cell phone in direct contact with the breast are subject to recall bias.

However, the unusual pattern of multifocal cancers and extensive DCIS occurring in areas of direct cell phone contact on the breast is noteworthy. Each patient had multifocal cancer, but the tumors were all clustered within the area of breast tissue directly underlying the cellular device, and nowhere else. Furthermore, from a pathological point of view, the morphological features of insitu and invasive ductal carcinomas are interesting. All of the carcinomas exhibited similar, if not identical, histology characterized by a mixture of tubular and solid patterns with identical nuclear morphology and grade. All were estrogen and progesterone positive but Her2 negative, luminal-type carcinomas. The ductal and lobular units away from the areas of cellular phone contact showed no significant histological changes. While the cancers appear to be centralized to the region of the breast exposed to the cellular devices, they still possess the ability to metastasize as evidenced by three patients in this series with lymph node metastasis and one with bone metastasis (Case 2). Although the numbers of reported cases here are too small to have a scientific conclusion, the findings are intriguing and support the notion that direct cellular phone contact may be associated with the development of breast carcinoma. 

There are fundamental differences between the available literature on cellular devices associated with cancer development and the four cases presented here. First and foremost, unlike the brain which is protected by the skull as well as a spatial distance from the cellular device, each patient here had direct contact between their device and their breast. The effect of EMR on tissues is directly related to the distance between the body and the source [2]. No study has yet to evaluate this direct effect. Second, the period of exposure was prolonged, over many years. Patients from earlier studies in general had a shorter duration of exposure to cellular EMR compared to those in our series. Lastly, it has been demonstrated that the effect of EMR on children can be several times higher than that of adults [22]. It is possible that the growing, dividing breast tissue that occurs during puberty may be particularly vulnerable to cellular phone EMR, accounting in part for at least two of the cases reported here (Cases 1 and 2). 

Cellular phone use continues to expand rapidly, especially among young adults. Until more data becomes available, efforts should be made to encourage cellular phone users to follow the recommendations of mobile device manufacturers and to avoid skin contact. Further research is urgently needed in this area. In our practice, we have started to incorporate frequency of cellular phone use and placement location into part of our routine patient-history documentation. Physicians should document this behavior and also inform their patients that, until sufficient safety data becomes available, prolonged skin contact with cellular devices should be avoided.
